# Atmosphere-Assisted FLASH Sintering of Nanometric Potassium Sodium Niobate

**DOI:** 10.3390/nano12193415

**Published:** 2022-09-29

**Authors:** Ricardo Serrazina, Luis Pereira, Paula M. Vilarinho, Ana M. Senos

**Affiliations:** 1 Department of Materials and Ceramic Engineering, CICECO—Aveiro Institute of Materials, University of Aveiro, 3810-193 Campus Santiago, Portugal; 2 CENIMAT-I3N, School of Science and Technology, FCT-NOVA, Universidade NOVA de Lisboa, Campus da Caparica, 2829-516 Caparica, Portugal

**Keywords:** atmosphere-assisted FLASH sintering (AAFS), low-temperature sintering, KNN, nanopowders, electrical conductivity

## Abstract

The request for extremely low-temperature and short-time sintering techniques has guided the development of alternative ceramic processing. Atmosphere-assisted FLASH sintering (AAFS) combines the direct use of electric power to packed powders with the engineering of operating atmosphere to allow low-temperature conduction. The AAFS of nanometric Potassium Sodium Niobate, K_0.5_Na_0.5_NbO_3_, a lead-free piezoelectric, is of great interest to electronics technology to produce efficient, low-thermal-budget sensors, actuators and piezo harvesters, among others. Not previously studied, the role of different atmospheres for the decrease in FLASH temperature (T_F_) of KNN is presented in this work. Additionally, the effect of the humidity presence on the operating atmosphere and the role of the compact morphology undergoing FLASH are investigated. While the low partial pressure of oxygen (reducing atmospheres) allows the decrease of T_F_, limited densification is observed. It is shown that AAFS is responsible for a dramatic decrease in the operating temperature (T < 320 °C), while water is essential to allow appreciable densification. In addition, the particles/pores morphology on the green compact impacts the uniformity of AAFS densification.

## 1. Introduction

FLASH is an electric field and current-assisted sintering technique that has been reported to dramatically decrease the sintering temperature and time of ceramics [1,2,3]. The direct application of an electric field to an insulator, along with external factors that promote electrical long-range conduction in the material (the temperature, sintering atmosphere, and others) result in a current increase during the FLASH process that is responsible for fast densification [3]. Most of the research has been performed in constant heating rate (C.H.R.) processes, where the conduction mechanisms of un-sintered pellets (green bodies) are thermally activated. 

In such cases, the FLASH temperature, T_F_, is defined as the temperature at which the material has sufficient conductivity to allow a current flow and FLASH sintering to occur. During the heating and simultaneous application of the electric field, three stages are identified: stage I, incubation, where the electric field and external heating are responsible for the nucleation and activation of conducting mechanisms, without significant densification occurring; stage II, when FLASH occurs, with a power spike and fast, abrupt densification; and stage III, or steady-state, when the pellet undergoes the remaining densification towards full density and the current is limited, avoiding total melting of the ceramic [3,4].

Apart from the thermally activated FLASH-sintering processes, Atmosphere-Assisted FLASH sintering, herein designated as AAFS, has been stated to reduce T_F_. Nanometric zinc oxide, ZnO, was FLASH sintered to 98% relative density at room temperature under a reducing humidified atmosphere of Ar/H_2_ + H_2_O [5]. Previously, this material had been FLASH-sintered in dry Ar/H_2_, however, at ≈120 °C [6]. The reducing atmospheres, such as the Ar and Ar/H_2_ mixtures, were described to increase the conductivity of ZnO at low temperature, first by decreasing the PO_2_ and second with an added effect of hydrogen interstitial defects working as shallow donors [6]. 

Additionally, ZnO was used to demonstrate a new, low-temperature sintering technique, FLASH-Cold sintering. In this case, not only is H_2_O added to the powders but also an external pressure and electric field are applied to promote sintering [7]. While AAFS and FLASH-Cold sintering are promising techniques, a clear understanding on the activation of conduction and respective sintering processes was not achieved.

Potassium sodium niobate, K_0.5_Na_0.5_NbO_3_ (KNN), a promising lead-free piezoelectric, was reported to FLASH sinter in Air at temperatures around 900 °C [8,9,10]. Moreover, AAFS allowed to densify KNN pellets below 300 °C in a humidified Ar atmosphere [11]. In this work, a high current density (60 mA/mm^2^) was used to promote densification at low temperature, and it was postulated that preferential melting through the particles’ surfaces was the consequence of current flow and water adsorption in the green pellet. Such melting would allow fast densification through the sliding of grain boundaries and viscous flow of the liquid, driven by the minimization of the compact’s surface energy [11]. Nonetheless, current localization was revealed with non-uniform densification reported.

If KNN is to be used as a lead-free piezoelectric for the replacement of market leader PZT (Pb(Zr_1−x_Ti_x_)O_3_), further understanding of the role of atmosphere for the low temperature FLASH sintering is needed. Knowledge on the conducting mechanisms and their effect on the densification during AAFS of KNN is needed, especially when reducing atmospheres as Ar and Ar/H_2_ are used. This will encourage process developments towards room temperature and the sustainable processing of lead-free piezoelectrics and other materials.

In this work, AAFS was used to sinter nanometric KNN powders, with different granulometry, for the establishment of the link between particle characteristics, FLASH temperature and operating atmosphere. The role of sintering atmosphere on T_F_ was studied using argon (Ar), a mixture of argon with hydrogen (Ar/H_2_) and Air. The role of water was assessed by humidifying the reducing gases. The activation energies for conduction during AAFS and Air FLASH were calculated and compared, establishing the mechanisms for conduction.

## 2. Experimental

Potassium Sodium Niobate, K_0.5_Na_0.5_NbO_3_ (KNN) powders with 99% purity were produced via a conventional solid-state reaction as previously reported [11]. After calcination, two dissimilar milling steps were considered for particle size control. A conventional ball milling (BM) step of 24 h, at 200 RPM in Teflon jars, using YSZ balls and ethanol as dispersant, produced the BM powder. 

In parallel, as-calcined powders were attrition milled (AM), with YSZ balls in ethanol, using a Teflon jar; however, in this case, at 700 RPM for 14 h, producing the AM powder. The powder morphology, size, structure and chemistry were characterized and presented in detail elsewhere [12]. In addition to the previously described characterization, the powder crystallite size was determined using X-Ray Diffraction (XRD, XPERT-PRO PANalytical, Almelo, The Netherlands) with a copper X-ray source, − Kα_1_ = 1.54060 $\overset{\cdot }{A}$ pattern, using a LaB_6_ standard for the calibration of the instrumentation broadening.

Both BM and AM powders were uniaxially (ca. 130 MPa) and isostatically (ca. 200 MPa) pressed into parallelepipedal-shaped pellets of ca. 7 × 5 × 2 mm^3^. To determine the pore size and distribution in green compacts, a mercury porosimeter was used (*Micromeritics*—*Autopore IV 9500*, 4356 Communications Dr, Norcross, GA 30093-2901, USA).

To study the FLASH-sintering process of compacts under different atmospheres, silver electrodes were painted in opposite faces and dried in ambient conditions for 1 h. This process guarantees good electrical contact between the KNN compacts and the platinum sheets. The powder compacts were placed in an alumina sample holder with a pushing rod to maintain the contact between the two opposite platinum electrodes, as reported previously [11]. 

To perform the AAFS process, an atmosphere-controlled furnace was used, and the reducing gases (argon or argon + 5 vol% hydrogen mixture, Ar and Ar/H_2_, respectively), were constantly fluxed (ca. 500 mL/min) into the sample’s holder system; a purging time of 30 min was executed prior to the heating and electric field application (in similar conditions to those previously reported [5,11]). 

Furthermore, to produce humidified atmospheres, the Ar and Ar/H_2_ gases were bubbled through a water-containing flask, (achieving a 100% relative humidity content on the environment, measured with a *DeltaOhm HD2717 sensor,* 35030 Caselle di Selvazzano, Italy) and flowed to the sample holder in the same conditions as dry atmospheres. For comparison, Air FLASH experiments were conducted. In this last case, only platinum sheets were used as electrodes, due to silver’s limited thermal stability. All FLASH-sintering experiments were conducted at a 10 °C/min heating and cooling rate with a constant electric field (*EPS HV 5006-400* DC power source) of 300 V/cm.

The conductivity (σ) of green KNN compacts was calculated from the direct measurement of the current flow through the system, which was limited to 20 mA/mm^2^. It is important to note that, in the present work, the current density was limited to 20 mA/mm^2^ to avoid the formation of broad hotspots and channeling of the electrical current as previously reported [11,13]. The furnace temperature, applied voltage and current were acquired using in-house developed hardware and software. To determine the activation energy for conduction (E_a_(σ)) of KNN pellets during stage I of FLASH, an Arrhenius representation of ln(σ) as a function of 1/*Tcalc* was considered. 

Here, *Tcalc* stands for the estimated specimen temperature calculated by the non-equilibrium development of the black-body radiation model from Raj [14], which is further developed in [15,16] and here presented in Equation (1) for the estimation of the sample temperature (*Tcalc_i_*) of two consecutive data points (i = 1 and 2). 

In Equation (1), *t* is the time; *m* is the initial mass of the pellet; *C_p_* is the heat capacity of KNN (800 J.Kg^−1^.K^−1^); *V* and *I* are the measured voltage and current, respectively; *k_SB_* is the Stefan–Boltzmann constant; and *T* is the furnace temperature. An emissivity of 1 was considered, and the radiant surface (*S_lat_*) was approximated to the four faces of the parallelepipedal pellet that were not in contact with the platinum electrodes. For sample temperature estimation, the heat dissipation by conduction/convection was not considered.

(1) $$Tcalc_{2}^{}=Tcalc_{1}^{}+\frac{t_{2}-t_{1}}{mC_{p}}\left[V_{1}I_{1}-k_{SB}\varepsilon S_{lat}(Tcalc_{1}^{4}-T_{1}^{4}\right]$$

Before and after sintering, all the ceramics were measured, and the geometrical apparent density, as well as the relative density considering a theoretical density of 4.5 g/cm^3^, was calculated. To evaluate the macroscopic appearance of AAFS KNN-sintered ceramics, an optical microscope was used (*LEICA EZ4HD,* Leica Microsystems (Switzerland) Ltd. Industry Division. Max Schmidheiny Strasse 201. CH-9435 Heerbrugg (Switzerland). For the microstructural analysis of powders, compacts and sintered ceramics, a field-emission scanning electron microscope (SEM, *Hitachi SU-70,* Hitachi ltd., Tokyo, Japan) at a 15 kV acceleration potential was used.

## 3. Results

### 3.1. Characterization of Powders and Compacts

Table 1 shows the characteristics of the produced KNN nanometric powders. Detailed analysis may be found elsewhere [12]. In the scope of the present work, it is important to note that BM and AM have significantly different features, due to their final milling step. 

In summary, BM is the coarser powder, which is shown by the average particle size determinations (either by specific surface area, laser diffraction or SEM imaging) but also confirmed by crystallite size determinations (from XRD)—Table 1. The crystallite size of both powders is approximately half of the particle size, indicating that particles are not monocrystalline. Additionally, the chemical composition (determined by the ICP) of powders reveals that the alkali ratios (K/Na and (K + Na)/Nb) agree with K_0.5_Na_0.5_NbO_3_ composition and that the contamination from YSZ balls (milling media) is residual for both cases [12].

**Figure 1:**
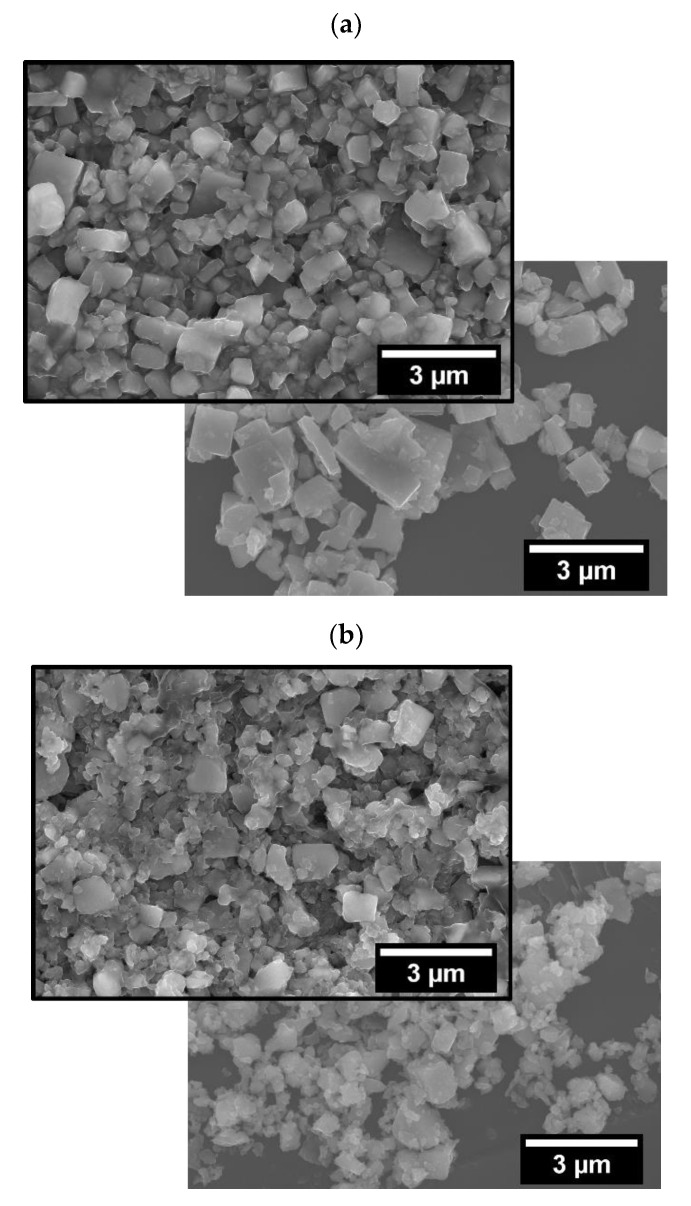
SEM micrographs of (**a**) BM and (**b**) AM loose powders, overlapped with the respective green compacts’ microstructure.

Figure 1 shows the micrographs of powders and respective compacts of (a) BM and (b) AM KNN. The smaller particle size of AM KNN is demonstrated, while a cuboid particle shape is revealed for both powders, which is typical of KNN. Table 2 shows additional KNN compact features. The geometrical green density, the porosity and the equivalent average pore size are presented, and the data shows that the coarser powder (BM) and the finer one (AM) present distinct packing characteristics. AM compacts have a slightly higher green density (determined geometrically) and lower porosity and average pore size (determined by porosimetry). 

Some discrepancy is observed between the geometric green relative density and porosity values—namely, in the AM compact. This is related to the lowest limit of detection of the porosimeter on tens of nanometer, which may result in an underestimation of the porosity values for finer pore-size distributions, as in AM, coupled with the underestimated values of geometrically measured densities. 

**Figure 2:**
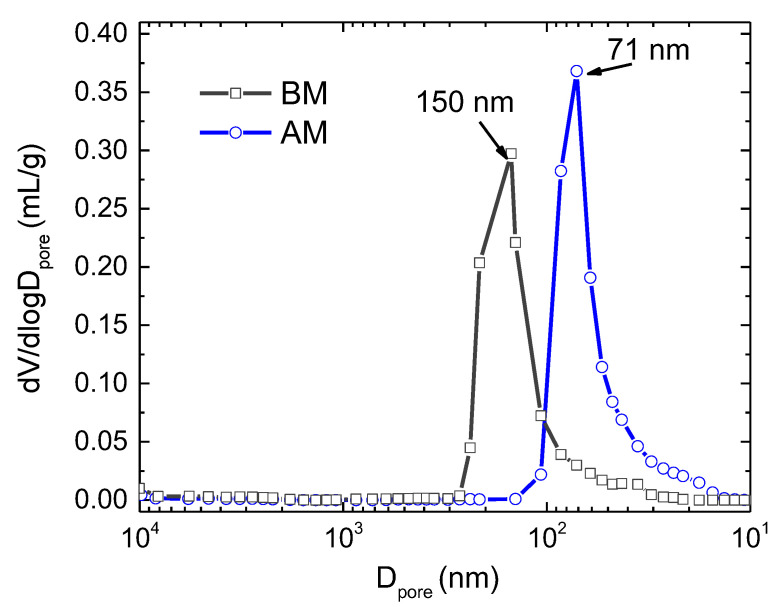
Differential volume intrusion per mass unit as a function of the pore diameter (D_pore_).

Table 2 also shows that the average pore size ($\bar{D_{\mathrm{pore}}}$) of AM compacts is ~71 nm, less than half of that of BM (150 nm), which is revealed in Figure 2. In this figure, the differential mercury volume intrusion as respect to the pore diameter for the KNN compacts is shown. It is clearly indicated that BM compacts present larger pore size (D_pore_), associated with a wider distribution of sizes (20–300 nm). In opposition, AM compacts show a sharper differential volume intrusion maximum (from 10–100 nm) with a lower average pore size. The lower limit of the pore-size distribution in AM is coincident with the detection limit of the porosimetry technique, as referenced above, and the average pore size value of ~71 nm can be, in fact, overestimated. However, this is not considered relevant for the present study.

In summary, the finer (AM) particle-sized powder resulted in green compacts with densities close to those of coarser BM powder compacts, but with a finer and narrower pore-size distribution. These observations indirectly indicate that the pore network (porosity channels) must be more uniformly distributed in the case of AM.

### 3.2. FLASH Sintering in Dry and Humidified Atmospheres

**Figure 3:**
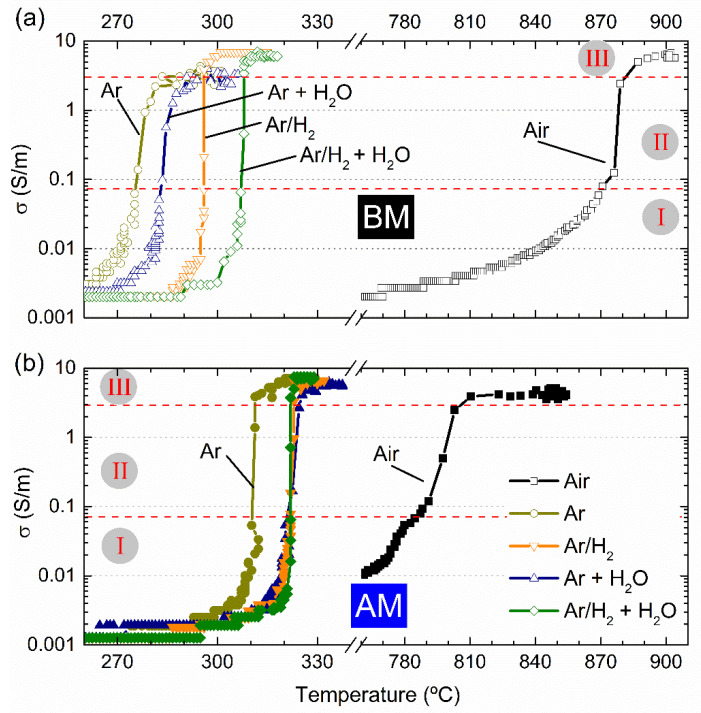
The in situ conductivity over temperature during FLASH experiments of (**a**) BM (open symbols) and (**b**) AM (closed symbols) compacts, under different atmospheres: Air (squares), Ar (circles), Ar/H_2_ (down triangles), Ar + H_2_O (up triangles) and Ar/H_2_ + H_2_O (diamond). Indications of FLASH stages (I, II and III) are given.

The electrical conductivity as a function of furnace temperature is represented in Figure 3 for BM and AM powders, in (a) and (b), respectively. Equal electrical and thermal conditions but different sintering atmospheres were used to FLASH sinter such compacts—namely, dry ones (Air, Ar and Ar + H_2_) and humidified (Ar + H_2_O and Ar + H_2_ + H_2_O). The data shows rather similar FLASH processes independently of the employed atmosphere; however, that is not the case for the FLASH temperature, T_F_. 

A clear and prominent dependence of T_F_ with the operating atmosphere is revealed. Table 3 summarizes the FLASH temperatures estimated from Figure 3. Each experimental condition was repeated at least once, and the standard deviation from the mean value of T_F_ was calculated when three or more repetitions were performed. Additionally, Table 3 gives information on the relative final density (ρ_sint_) of the BM and AM sintered compacts.

A first analysis on BM compacts revealed that the use of Ar atmosphere allows a T_F_ decrease from 870 °C (Air) to 276 °C. If a hydrogen-containing dry atmosphere is used (Ar/H_2_), the FLASH temperature is slightly increased to 295 °C. A similar tendency is observed when humidified reducing atmospheres are employed, with Ar + H_2_O and Ar/H_2_ + H_2_O giving slightly higher T_F_ (284 and 306 °C, respectively) than the correspondent dry atmospheres.

Previously reported for ZnO [6] and KNN [11], the use of low oxygen content (reducing) atmospheres dramatically decreases T_F_, which is related to the defect chemistry of materials, a topic discussed later on. Additionally, when hydrogenized [6] and/or humidified [5] atmospheres are used to FLASH sinter ZnO, the T_F_ is further decreased (ultimately, to room temperature [5]). 

However, in opposition to that, both hydrogenized (Ar/H_2_) and humidified atmospheres (Ar + H_2_O and Ar/H_2_ + H_2_O) used in AAFS of KNN faintly increased T_F_, in comparison with the correspondent non-hydrogenized and/or dry atmospheres. These observations must have a relationship with the interaction of different gases and humidity with the material, which is not known yet.

In parallel, Table 3 and Figure 3 reveal that AAFS of BM compacts resulted in low final densification (ρ_sint_). For compacts AAFSed under Ar, Ar/H_2_ and Ar/H_2_ + H_2_O, the final density is not increased when compared with the un-sintered compacts, meaning that sintering did not occur. Nonetheless, the densification of compacts FLASH sintered in Ar + H_2_O, is already appreciable, leading to specimens with 74% of the relative density.

Even though water’s role on AAFS is not yet well understood, it was demonstrated to be associated with the achievement of high density for ZnO (98%) by a process based on increased mass transport and consequent greater densification [5]. In the case of KNN, water shows a promising effect towards the increase in density for AAFS in an Ar + H_2_O atmosphere.

When comparing BM with finer particle AM compacts, Table 3 shows that the final densities of both are similar, regardless of the operating atmosphere during FLASH. However, that is not the case for T_F_, as shown in Figure 3 and Table 3. For experiments in Air, the T_F_ of AM is decreased when compared with that of BM (785 and 870 °C, respectively). This is related to the effects of the particle-size reduction and correspondent particle-contact-density increase, as previously reported and discussed [12].

When AAFS is employed in AM compacts, a general observation reveals that a similar trend is obtained as in BM with reducing atmospheres contributing for a strong decrease in T_F_. In a parallel link, the use of hydrogenized and humidified atmospheres also resulted in slight T_F_ increases. Additionally, Figure 3 and Table 3 reveal that, while in Air, the T_F_ is decreased for AM powders when compared with BM ones, the use of reducing atmospheres promoted the opposite tendency, with AM compacts AAFSed in Ar and Ar/H_2_ presenting a higher T_F_ than the BM ones.

The fact that the decrease of particle size augments the density of particle-to-particle contacts per volume unit explains the decrease of T_F_ in AM compacts compared with BM ones when Air FLASH is performed. However, it does not remain valid to explain the opposite tendency observed during AAFS. As presented before, Table 2 and Figure 2 show that in green BM compacts the pores are coarser than in AM.

Coarser porosity channels should increase the gas permeability in the green pellets and a more pronounced effect of its interaction with the powder’s surfaces. This effect must overlap the effect of the lower particle contact density in the compacts with coarser powder (BM) when compared to finer particle ones (AM), explaining its lower T_F_ during AAFS. The increase in T_F_ when using Ar/H_2_ in comparison with simple Ar remains a topic to be clarified.

Despite the detailed discussion on T_F_ dependencies, Figure 3 also suggests that the conductive behavior of KNN powders FLASH sintered in Air or in reducing atmospheres is different for both BM and AM compacts. While a slow increase in σ with temperature occurs in Air, the transition from low conductivity (0.001 S/m) towards the FLASH event (σ ≳ 0.07 S/m)—stage I to stage II—during the AAFS processes is faster, identifiable by the minor quantity of data points in Figure 2. In parallel, Table 3 reveals that the final density is affected by the AAFS in non-oxidizing atmospheres. Therefore, the mechanisms that promote FLASH must be different than those operating for Air FLASH, which is why they were investigated.

**Figure 4:**
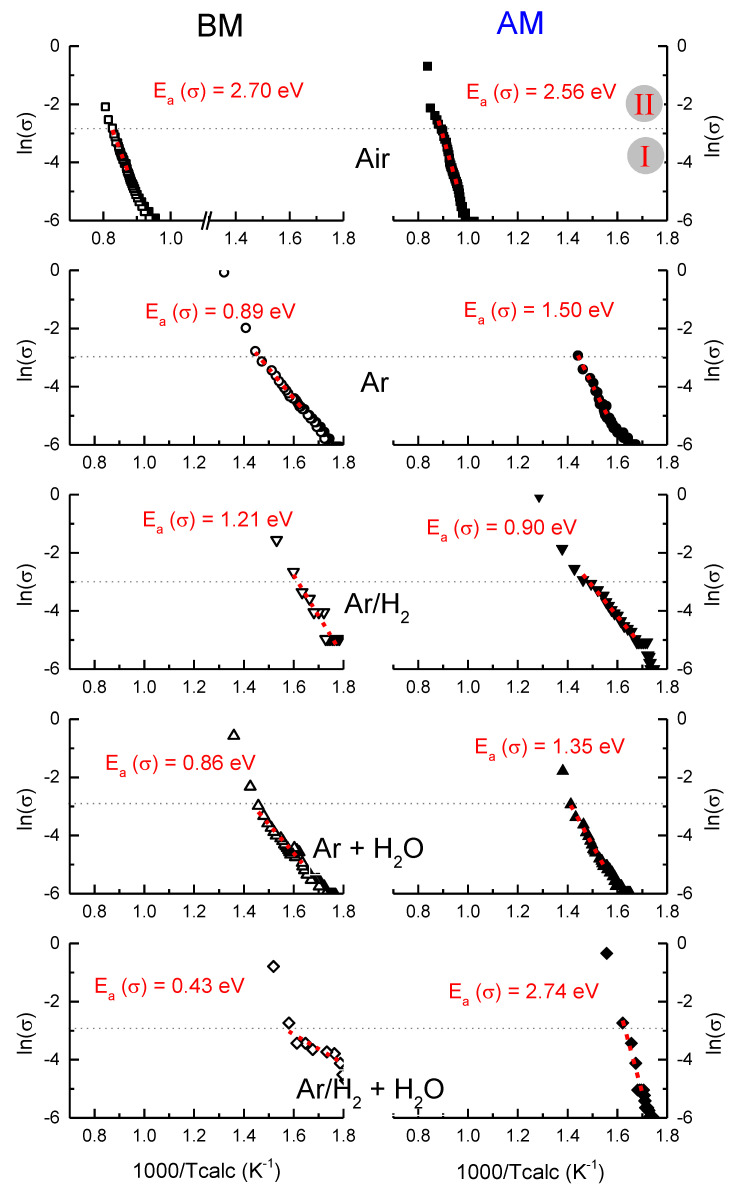
Arrhenius plot of conductivity for BM (**left-hand**, open symbols) and AM powders (**right-hand**, closed symbols) when FLASH sintered in atmospheres of Air (squares), Ar (circles), Ar/H_2_ (down triangles), Ar + H_2_O (up triangles) and Ar/H_2_ + H_2_O (diamond). The activation energy for the stage I of FLASH is calculated from the plots and shown for each case.

Figure 4 shows the Arrhenius representation of the electrical conductivity using the estimated specimen temperature (Tcalc) obtained from the non-equilibrium adaptation of the Black Body Radiation model [15,16], in which dT/dt is different from zero during stage I of FLASH. In a general observation, one can state that the dependency of the conductivity on the temperature is similar to both BM and AM compacts when FLASH sintered in Air. However, that dependence is different when non-oxidizing AAFS is performed.

In detail, the maximum calculated temperature achieved during stage I of FLASH in Air and Atmosphere-Assisted is significantly different, independently of the powder (BM and AM). In the first case (Air), stage I is completed at 1000/Tcalc ≈ 0.8 K^−1^, or 977 °C. Instead, for the AAFS in reducing conditions, the stage I maximum temperature is roughly independent of the atmosphere and occurs at 1000/Tcalc ≈ 1.4 K^−1^, or 441 °C. Exceptions are made for both compacts in Ar/H_2_ + H_2_O in which the temperature at the end of stage I is roughly 350 to 390 °C. 

There is more than a 100 °C difference between humidified-hydrogenized-Argon and the remaining AAFS atmospheres and about a 500 °C difference between the later and Air experiments. Moreover, the transition regime, associated with stage II, is also dissimilar, as previously stated (Figure 3). A significantly larger number of data points, acquired constantly with a time interval of 1 s in all experiments, was recorded for Air FLASH. In contrast, a fast transition occurs for AAFS during stage II, with only a few points being recorded. 

This was especially evident in the Ar/H_2_ + H_2_O atmosphere. These conditions together induce a smaller generation of heat by Joule effect, observed clearly by the lower calculated temperature during stage I for AAFS when compared with Air FLASH. One can say that thermal runaway phenomena is not ruling in AAFS of KNN, thereby, hindering the densification. 

As indicated by J. Nie and co-workers [5], in the water-assisted FLASH sintering of ZnO, densification only occurs when the estimated sample temperature is above a threshold (1100 °C, in that case). For AAFS of KNN, we postulate that the minimum estimated temperature for the transition between stage I and II must be close to 1000 °C (Air conditions) so that high densification is achieved.

In parallel, the apparent activation energy for conduction, E_a_(σ), during stage I was estimated from the Arrhenius representation of conductivity over the estimated temperature and is also shown in Figure 4. The results confirm the thermally activated processes for all the pellets and atmospheres. However, differences in the apparent E_a_(σ) as a dependence of the process were found. In detail, when FLASH sintered in Air, BM and AM, the pellets presented E_a_(σ) between 2.6 and 2.7 eV. In contrast, when reducing atmospheres were used to perform AAFS, E_a_(σ) was between 0.9 and 1.5 eV, independently of the powder and atmosphere. The exception was again the Ar/H_2_ + H_2_O atmosphere, in which BM compacts presented an activation energy of 0.43 eV for conduction, while for AM ones, E_a_(σ) = 2.74 eV.

Studies on the DC conductivity of the perovskite-structured La-doped BFO indicate that, if the condition 0.2 < E_a_(σ) < 0.45 eV is satisfied, a conduction mechanism dominated by p-type polaron hopping occurs [17,18,19]. In parallel, K_0.5_Na_0.5_NbO_3_ has been reported to present p- or n-type behavior when sintered in Air or N_2_, respectively. Moreover, high dielectric losses were found for the ceramics sintered under a reducing N_2_ atmosphere, which was attributed to a higher concentration of oxygen vacancies, $V_{O}^{{}^{\circ}{}^{\circ}}$, in such ceramics [20]. In low PO_2_ atmospheres, the formation of $V_{O}^{{}^{\circ}{}^{\circ}}$ is facilitated (Equation (2)). As Nb^5+^ is a d-cation, it would accommodate the excess of electrons [20]; however, it is possible that such excess of electrons also contributes to the conduction.

(2) $$O_{O}^{\times }\;\to \frac{1}{2}O_{2}^{}\left(g\right)+{\;V}_{O}^{{}^{\circ}{}^{\circ}}+2\;e^{\prime }$$

Complementarily, the activation energies for conduction in ferroelectric perovskites reported between 0.4 and 1.2 eV have been associated with charge transport by ionized oxygen defects [21,22]. This was confirmed previously for KNN ceramics and single crystals [12,23]. On the other hand, ionic-based conducting mechanisms have been reported in KNN single crystals to present activation energies higher than 1.2–1.3 eV [23].

The data reported in Figure 4 suggests that the conducting mechanism during the Air FLASH sintering of dry KNN powders is suitable with ionic-based conduction because of the condition E_a_(σ) > 1.2 eV. This conduction process can be interpreted as intrinsic conduction. 

On the other hand, the movement of thermally activated ionized $V_{O}^{{}^{\circ}{}^{\circ}}$ appears to be the ruling mechanism in AAFS because the condition 0.4 < E_a_(σ) < 1.2 eV is satisfied. Therefore, extrinsic conduction occurs. Once again, exception made for AM compact in Ar/H_2_ + H_2_O, where ionic conductivity seems to take place, which is an unexpected result. 

The limited number of points in the Arrhenius plots of Figure 4 and deviations from the assumptions assumed in Equation (1)—namely, the contribution of the heat dissipation by conduction/convection in the sample temperature estimation of AAFS samples increases the error associated to the determined activation energy values. In the case of Ar/H_2_ + H_2_O, there is a higher fluctuation of the conductivity values, probably coming from the lack of uniformity of the humidity in the atmosphere and which is believed to be the reason for the unexpected, calculated differences on the activation energies values; however, this topic requires further investigation.

It is suggested that, in AAFS, there was an increase in the concentration of $V_{O}^{{}^{\circ}{}^{\circ}}$ at low temperature promoted by the low partial pressure of oxygen, facilitated by the polaron hoping and excess of electrons that allow conduction at T < 330 °C; in contrast, in Air FLASH, ionic conduction is activated. The presence of oxygen does not allow a relevant contribution of oxygen vacancies for the conduction. In that case, a significant local heating by Joule effect (thermal runaway) promotes the partial melting of particles’ contacts and the following densification of KNN compacts [9,11]. This process does not occur in AAFS due to the significantly lower furnace temperature and higher concentration of conducting defects (extrinsic) hindering the Joule heating generation and thermal runaway process.

Figure 4 shows that similar conducting mechanisms are obtained for each powder (AM and BM), if the same type of atmosphere is considered. This observation indicates that, independently of the particle contact density and pore morphology, the conducting mechanisms during FLASH are the same for each atmosphere, and T_F_ is changed only by the interaction of the gas and moisture with the KNN compacts. These results contrast with the observations in ZnO [5,6], where AAFSF led to high densification at room temperature (98% of relative density).

**Figure 5:**
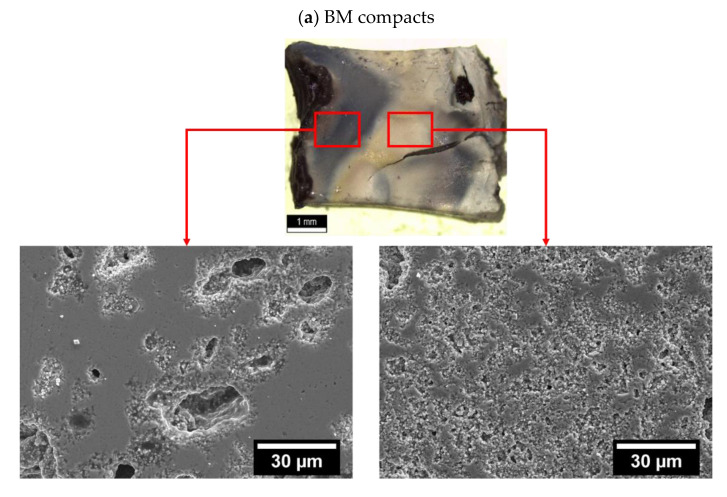

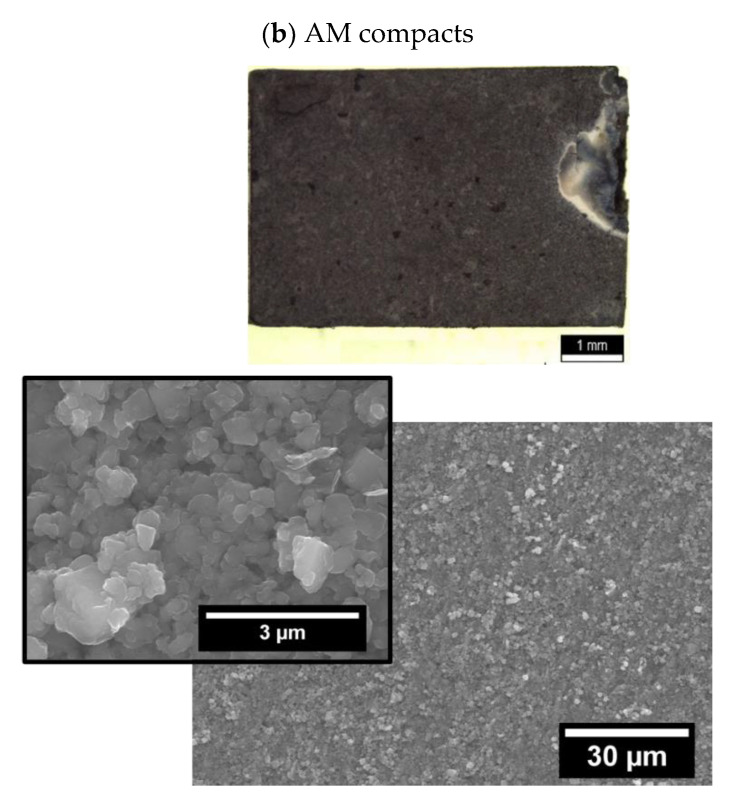
Photographic report and microstructure of (**a**) BM and (**b**) AM compacts AAFSed under Ar + H_2_O showing a lower magnification image overlapped with a higher magnified one.

The analysis of operating conducting mechanisms during AAFS may lead to the conclusion that the initial particle size and consequent pore morphology do not affect the FLASH-sintering process. However, the final density of AAFSed KNN is dependent on the atmosphere and the presence of water (Table 3). On the one hand, when FLASH sintering both BM and AM compacts in Air, the whitish color of compacts was found to be kept on the sintered ceramics. On the other hand, that is not the case of AAFS. While a uniform dark color was identified in all AAFSed AM compacts, BM pellets appeared with dark non-uniform localized areas. As an example, the final macroscopic appearance and respective microstructures of ceramics AAFSed in Ar + H_2_O of BM and AM powders are shown in Figure 5, (a) and (b), respectively. Dark colors in KNN ceramics are typically associated with the presence of oxygen vacancies. 

Hence, by direct observation of Figure 5, it is possible to infer that AM pellets present a significantly more uniform distribution of these defects, when compared with coarser BM powders, independently of the operating atmospheres during AAFS. As revealed in (a), the localized darker areas in BM ceramics are associated with higher density as a consequence of current localization and hotspots [11]; on the other hand, white areas are not well densified. In opposition, Figure 5b shows a uniform ceramic with a homogeneous microstructure; nonetheless, the microstructure inset reveals a still low final density, in accordance with Table 3.

The dissimilarities in pore size and distribution in AM and BM compacts may explain their different AAFS behavior (Table 3 and Figure 3) and consequent appearance and microstructure (Figure 5). The atmosphere interaction with the powders is achieved through the pore channels available for gas adsorption. Therefore, the coarser, less uniformly distributed pore channels in BM compacts are prone to promote current localization when a FLASH-sintering atmosphere-dependent process is performed, contributing to the hotspot formation (Figure 5) in accordance with previous work [11]. Furthermore, the finer and more uniformly distributed pores in AM explain the limit conditions found for T_F_, where the use of reducing dry and humidified atmospheres (except for Ar) gives approximately the same FLASH temperature (Figure 3: T_F_ = 319 to 322 °C). 

As the gas interaction with the powder is limited by the amount of that gas (and humidity) that can be adsorbed in the particles through pores, a smaller pore size induces a limited concentration of gas to interact with the particles. However, this interaction is achieved uniformly through the compact, avoiding current localization and hotspots. The validity of such affirmation is ensured because there is no pressurization of gases during AAFS. Therefore, it is suggested that, while the difference in E_a_(σ) of Air and AAFS might explain the low densification obtained on the latter case, the pore-size distribution has a fundamental role to promote a uniform interaction of gases and humidity during AAFS. 

Regarding the final density of AAFSed ceramics, an increase in density (from the green state) was observed for both AM and BM compacts sintered in reducing dry atmospheres; on the other hand, when humidified atmospheres were used—namely, argon—an already appreciable densification (~74 to 75%) was found. However, hotspots occurred in coarser BM powder compacts, while a uniform atmosphere interaction with AM compacts was found. Despite that the conducting mechanisms may be similar for wet/dry and simple/hydrogenized reducing atmospheres, the different T_F_ and ρ_final_ of AAFSed ceramics indicate that water plays a role in increasing the densification.

## 4. Conclusions

AAFS was proven to be a promising technique to reduce the T_F_ of KNN; however, the densification is limited. A high concentration of conductive defects at low temperature promotes conduction. However, this does not allow sufficient heat to be generated for densification, i.e., the occurrence of thermal runway. Low energy oxygen vacancy movement and/or recombination is the ruling mechanism in AAFS, while ionic conductivity commands the FLASH of KNN in Air.

A relevant improvement of the final densification obtained by AAFS was found by using a humidified argon atmosphere and a nanometric particle size, leading to the highest density reported for AAFS with microstructural homogeneity. The gas interaction with KNN is mandatory to reach such homogeneity, which was shown to be a consequence of the pore size and distribution, which is, ultimately, a consequence of the particle size and morphology.

## Figures and Tables

**Table 1:** KNN powder characteristics. Equivalent particle diameter (D_BET_) calculated from the specific surface area determination, average particle size from laser diffraction (D50-laser) and SEM images (D50-SEM), and crystallite size determined from the XRD and composition accessed by ICP (comprising alkali rations and Zr contamination). Adapted from [12].

	Particle Size			Crystallite Size	Composition (ICP)		
Powder	D_BET_ (nm)	D50-Laser (nm)	D50-SEM (nm)	D-XRD (nm)	K/Na	(K + Na)/Nb	Zr (at%)
**BM**	350	235	350	~100	1.0 ± 0.1	1.1 ± 0.1	0.10 ± 0.1
**AM**	171	204	210	~50			

**Table 2:** KNN compact characteristics. The green (geometric) relative density (ρ_green_); porosity and equivalent average pore size ($\bar{D_{\mathrm{pore}}}$ ) determined by mercury porosimeter.

Compact	ρ_green_ (%)	Porosity (%)	$\bar{D_{pore}}$ (nm)
**BM**	63 ± 2	33	150
**AM**	65 ± 2	28	71

**Table 3:** The FLASH temperature (T_F_) and sintered relative density (ρ_sint_) of KNN compacts in different atmospheres under a 300 V/cm electric field and 20 mA/mm^2^ current density limit at a constant heating rate of 10 °C/min.

KNN Powder	Atmosphere	T_FLASH_ (°C)	ρ_sint_ (%)
**BM**	Air	870 ± 5	92
	Ar	276 ± 3	65
	Ar/H_2_	295	61
	Ar + H_2_O	284 ± 4	74
	Ar/H_2_ + H_2_O	306	61
**AM**	Air	785 ± 6	89
	Ar	309 ± 4	72
	Ar/H_2_	320	63
	Ar + H_2_O	319 ± 4	75
	Ar/H_2_ + H_2_O	322	63
